# Community Attitudes toward Mass Drug Administration for Control and Elimination of Neglected Tropical Diseases after the 2014 Outbreak of Ebola Virus Disease in Lofa County, Liberia

**DOI:** 10.4269/ajtmh.15-0591

**Published:** 2016-03-02

**Authors:** Joshua Bogus, Lincoln Gankpala, Kerstin Fischer, Alison Krentel, Gary J. Weil, Peter U. Fischer, Karsor Kollie, Fatorma K. Bolay

**Affiliations:** Infectious Diseases Division, Department of Medicine, Washington University School of Medicine, St. Louis, Missouri; Liberian Institute for Biomedical Research, Charlesville, Liberia; London School of Hygiene and Tropical Medicine, London, United Kingdom; Ministry of Health, Monrovia, Liberia

## Abstract

The recent outbreak of Ebola virus disease (EVD) interrupted mass drug administration (MDA) programs to control and eliminate neglected tropical diseases in Liberia. MDA programs treat entire communities with medication regardless of infection status to interrupt transmission and eliminate lymphatic filariasis and onchocerciasis. Following reports of hostilities toward health workers and fear that they might be spreading EVD, it was important to determine whether attitudes toward MDA might have changed after the outbreak. We surveyed 140 community leaders from 32 villages in Lofa County, Liberia, that had previously participated in MDA and are located in an area that was an early epicenter of the EVD outbreak. Survey respondents reported a high degree of community trust in the MDA program, and 97% thought their communities were ready to resume MDA. However, respondents predicted that fewer people would comply with MDA after the EVD epidemic than before. The survey also uncovered fears in the community that EVD and MDA might be linked. Respondents suggested that MDA programs emphasize to people that the medications are identical to those previously distributed and that MDA programs have nothing to do with EVD.

## Introduction

Liberia is a post-conflict country in West Africa with a population of approximately 4.3 million. Like many other countries in sub-Saharan Africa, Liberia is endemic for a number of neglected tropical diseases (NTDs) that disproportionately affect poor populations in rural areas. Liberia's national NTD control and elimination program prioritizes onchocerciasis, lymphatic filariasis (LF), schistosomiasis, soil-transmitted helminth infections (STH), leprosy, and buruli ulcer, and it has only recently been scaled-up to reach all of the country's at-risk population. The program is using mass drug administration (MDA) with albendazole and ivermectin to eliminate LF and onchocerciasis.^1^ The national LF program conducted its first two annual MDA campaigns with albendazole and ivermectin in 2012 and 2013. The program achieved 100% MDA geographic coverage with albendazole and ivermectin for LF and ivermectin for onchocerciasis in all 13 and 15 endemic counties, respectively.^2^ The national NTD program director reported treatment coverage for both LF and onchocerciasis was approximately 82% in 2012 and 2013.

The Death to Onchocerciasis and Lymphatic Filariasis (DOLF) project has conducted an integrated NTD research project together with the Liberian Institute for Biomedical Research (LIBR) and the Liberian Ministry of Health and Social Welfare since 2012. The project compares the impact of annual and semiannual MDA with ivermectin, albendazole, and praziquantel on LF, onchocerciasis, STH, and schistosomiasis in 32 villages that are distributed around Foya town, the capital of Foya District in Lofa County.

Liberia is one of three West African countries that were severely affected by an outbreak of Ebola virus disease (EVD) that started in Guinea in late 2013. On March 30, 2014, the first two cases of EVD in Liberia were confirmed in Foya town in Lofa County, near Liberia's borders with Guinea and Sierra Leone.^3^ Lofa County went on to become an early epicenter of EVD in Liberia. A peak of 133 patients were admitted to the Ebola treatment unit in Foya during the week of August 16, 2014.^4^ By the end of the outbreak, a total of 332 confirmed cases and 451 confirmed or probable EVD deaths were reported in Lofa County.^5^ However, the health impact of the outbreak extended beyond cases of EVD, because it drained resources away from other essential health programs. As the health system became exclusively focused on combating the outbreak, bed nets for malaria prevention, MDA for NTDs, and vaccines were not delivered, and community health workers were seconded to the front lines of outbreak control. This indirect impact of EVD on public health services in Liberia, Sierra Leone, and Guinea is believed to have caused more deaths than EVD itself. For example, one study estimated that the disruption of malaria control and care programs may have resulted in an additional 3.5 million untreated malaria cases and 10,900 malaria-attributed deaths in 2014 in Liberia, Guinea, and Sierra Leone.^6^

Now that the outbreak is mostly under control and isolated to small geographical hotspots in Guinea and Sierra Leone, it is important that these countries' health systems resume public health programs that were suspended during the EVD epidemic. However, there is evidence that communities' attitudes toward the health system were altered by the EVD outbreak. During the outbreak, there were many reports of hostility toward health workers and even attacks on them, because some people feared that health workers were responsible for spreading EVD.^7^,^8^ An improved understanding of communities' attitudes regarding public health personnel and programs after the EVD outbreak may improve chances for smooth resumption of these public health programs. Therefore, the primary purpose of this study was to assess community leaders' knowledge and attitudes regarding resumption of MDA for NTDs after the EVD epidemic. We also aimed to identify how families and communities in the study sites were affected by the EVD outbreak. Finally, the survey provided an opportunity to solicit feedback from the communities themselves about how best to reengage their populations in MDA and research activities. Information from this survey helped us to restart the MDA study, and it may provide lessons for other public health programs that were suspended during the EVD outbreak.

## Methods

### Study sites.

**Figure 1.**
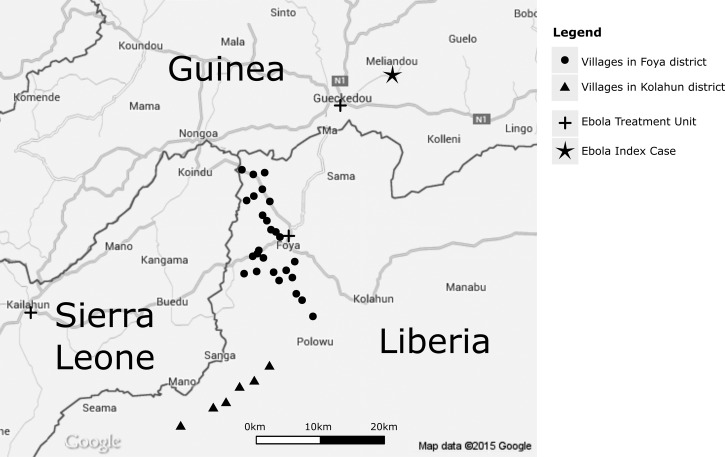
Map of the study area in northwestern Liberia.

The study was conducted in 32 DOLF study villages around Foya town in Lofa County in northwest Liberia (Figure 1

). Of these study villages, 26 were in Foya District and six were in Kolahun District. These districts are located near the borders of Sierra Leone and Guinea, and many of the residents in this area frequently cross these borders in the course of their daily activities. The districts are endemic for onchocerciasis, LF, STH, and schistosomiasis.^9^ The baseline assessment of parasite infection rates and intensities was completed in September 2012. MDA (albendazole, ivermectin, and praziquantel) was distributed in December of that year and again in June 2013 in the villages that were assigned to semiannual MDA.

The 12-month follow-up parasitological survey of all study villages started in January 2014. This included collection of blood and stool samples. The survey was about two-thirds complete by the end of March when Liberia reported the country's first case of EVD. DOLF management immediately suspended all research activity in this area because of safety concerns. The DOLF team at the LIBR was unable to return to Foya during the outbreak. Therefore, the DOLF parasitological survey was not completed and MDA was not provided in 2014 in our study area or anywhere in Liberia. This study was performed in early April 2015 as the LIBR was preparing to provide MDA that had been postponed because of the EVD outbreak.

### Questionnaire and survey procedure.

The survey questionnaire included questions related to trust and community compliance with MDA and parasitological examinations. It also asked participants to describe how the EVD outbreak had affected their families and communities. It was designed to take 15–20 minutes and it included a mixture of closed- and open-ended questions. Several iterations of the questionnaire were pretested in Monrovia and St. Louis to assess its duration and clarity.

The survey team included the project coordinator from LIBR, two local community health workers who provided translation services, and two members of the project team from Washington University in St. Louis. Four of the five survey team members had visited the study villages before the EVD outbreak and knew some of the residents. Villages were notified that the team would be coming so that the village chief and other leaders would be available. After the leaders and other community members were assembled, the survey team members introduced themselves, explained the reason why the MDA and research teams had not returned in 2014 as promised, and explained the purpose of the survey. Each community was asked to nominate three to five leaders who could speak on behalf of their village to participate in the survey. Communities were asked to nominate at least one youth leader and one female leader for the study to include a variety of perspectives. Survey participants included village chiefs, religious leaders, elders, and members of women's councils.

Participants were informed that their participation was voluntary and that they were free to stop the survey at any time or skip questions that they did not want to answer. The research was conducted as a part of the DOLF study that operates under ethical approval from institutional review boards at the University of Liberia-Pacific Institute for Research and Evaluation and at Washington University.

The survey team split into two groups to conduct the survey. One group conducted the interview in English for those who spoke the language well. For those who could not communicate in English, a second group conducted the interview through a translator fluent in either Kissi or Bandi, the main languages spoken in Foya and Kolahun districts, respectively. Responses were collected on laptop computers using EpiInfo 7 (Centers for Disease Control and Prevention, Atlanta, GA) and paper records were also prepared as a backup. The interviews were audio recorded with the permission of participants.

### Survey analysis.

After the survey was completed, two survey team members read through the responses to open-ended questions and identified the emerging categories that encapsulated the themes represented in the participants' responses. The two survey team members then independently back-coded the open answers using these categories. To achieve inter-rater reliability, the coding results were compared, and the two coders came to a consensus when initial coding results disagreed. In some cases, the audio recordings were used to verify the meaning of responses when the written answers were unclear. Categorical variables were compared between groups using a χ^2^ test or Fisher's exact test when appropriate. Analyses were performed using SPSS 22 (IBM Corporation, Armonk, NY).

## Results

### Survey participants.

A total of 140 community leaders were interviewed in 32 DOLF study villages in Lofa County (six in Kolahun District and 26 in Foya District) (Table 1). Of the survey participants, 74% were male (Table 2). The mean age of participants was 47 years (SD = 17).

### Community knowledge and trust in MDA.

Survey participants were generally well informed about MDA. The majority of respondents knew the purpose of MDA, and in an open-ended question, 124 (89%) were able to correctly name at least one infection or related symptom treated by MDA (filariasis, onchocerciasis, schistosomiasis, or intestinal worms). The survey respondents reported that this information was well known in their communities. When asked how many people in their village understood the purpose of MDA, 128 (91%) selected either “plenty of people” or “everyone.”

Respondents expressed a high degree of trust in the MDA program. Of the 131 people who remembered the last MDA and were present in the village at that time, 130 said that they thought the community trusted the treatment program. Trust in the program came overwhelmingly from the respondent's positive experience with treatment and feeling that the medicine was improving health generally (*N* = 118, 90%) or specifically improving symptoms related to onchocerciasis (*N* = 22, 17%) or schistosomiasis (*N* = 6, 5%). Other answers included that the village knew and recognized the people who provided the MDA from previous treatments or programs and therefore trusted them (*N* = 10, 8%), no one suffered unexpected side effects (*N* = 8, 6%), and the village had been educated about the medicine and therefore was ready to trust it (*N* = 7, 5%).

### Perceived and expected MDA coverage.

The village leaders that we spoke with perceived past MDA coverage as high. We asked survey participants how many people in the community they thought had swallowed the medicine during the last MDA using the following scale: 0 = no one, 1 = few people, 2 = some people, 3 = plenty of people, and 4 = everyone. Most felt that plenty of people (*N* = 84, 60%) or everyone (*N* = 50, 36%) in the town swallowed the medicine.

Most participants thought that their communities were ready to resume MDA (*N* = 130, 93%), and an equal number responded that they were willing to take the medicine if it were delivered “today.” However, when asked how many people they thought would swallow the medicine if it was delivered today, many survey participants expected fewer people would comply. A sign test was used to evaluate the direction of change in estimated coverage scores between the past MDA and MDA that was hypothetically delivered today. The results showed that when asked about MDA if it were delivered today, a significantly greater number of respondents lowered their coverage estimate relative to their prior experience (*z* = −5.207, *P* < 0.001; Table 3). On average, interviewees in Kolahun District lowered their coverage estimates more so than those in Foya District. The arithmetic mean coverage score for past MDA in Kolahun was 3.44 (median = 3). The expected mean coverage score was only 2.36 (median = 2) if the medicine was delivered today. The difference between scores was smaller in Foya, where the median was unchanged at 3 and the means were 3.29 and 2.83, respectively.

Some respondents explained their reasons for expecting that fewer people would comply with MDA. They explained that if MDA were delivered today it would be hard to get people to participate in the program since there had been no advance warning. It is possible that the fall in expected coverage may be partly explained by the wording of the question that led some respondents to consider the naturally lower levels of MDA coverage associated with a surprise MDA distribution. We continued to ask the question the same way throughout the study, though, when asked to clarify, we explained that we would inform the community on the purpose of the medicine before it would be administered.

### Suggestions for improving MDA coverage.

Village leaders suggested that more community education would be necessary to improve MDA coverage. The three most common themes in answers to an open-ended question were the following: 1) more information or education needs to be provided about MDA in advance of the distribution (*N* = 112, 80%), 2) reassure the community that the treatment is the same as before (*N* = 34, 24%), and 3) explain that MDA has nothing to do with EVD (*N* = 24, 17%). The frequency of these suggestions varied by district. People in Kolahun were less likely to suggest providing more information before MDA begins (χ^2^[1] = 4.795, *P* = 0.029) but more likely to mention that it was necessary to explain that MDA is not related to EVD (*P* = 0.049; Table 4).

In total, there were 52 (37.1%) people who suggested that we reassure communities that the treatment was the same as before or that MDA has nothing to do with EVD. All of these people mentioned fears within their communities about a potential link between EVD and the MDA program, namely that people involved in the EVD response might spread the disease or that the medicines themselves might cause EVD. The following quotes illustrate the fears expressed by community leaders:

There were stories that there were vaccines given to children who died around Ebola time. Come back with the same people and same drugs that helped us before and we would not fear so much and we would take them. (Female, 43 years old)

Educate them that the medicine is to sustain health and that the tablet is not killing them. Educate them that it is not Ebola medicine. (Male, 25 years old)

Everybody is scared of this Ebola business. Now people are afraid of the medicine. You need to tell the people that this medicine will help you to get well, it is filarial medicine, not Ebola medicine. (Male, 45 years old)

Most community leaders were willing to participate in future education campaigns. All but one answered that they would promote MDA. Those who were willing to promote MDA were asked to explain their motivation. The two most common themes were that the “medicine is good for the community” (*N* = 111, 79%) and that they wanted to serve their community (*N* = 29, 21%). An emphasis on communal benefits and duty were common in many statements such as “I want my community to be well,” “we can promote it so that the town can develop,” or “it is my duty.” Men were significantly more likely to mention service to community, with only two women citing it as a motivation (χ^2^(1) = 7.177, *P* = 0.007). Trust in the team that delivers MDA was the third most common motivation for promoting MDA. This was an especially important factor motivating women's interest in promoting MDA, as they were significantly more likely to mention this than men (*P* = 0.03).

### Expected participation in parasitological surveys.

Community leaders expected high levels of participation in future parasitological monitoring and evaluation surveys. Among the leaders themselves, 137 (98%) said that they personally would be willing to continue participating in planned examinations next year. Respondents were asked in an open-ended question what issues researchers could expect to face when they return to the community. According to 40 (29%) respondents, researchers should expect more people to participate in the examinations than before the EVD epidemic, while 17 (12%) respondents expected fewer people would participate due to fears from EVD. Some respondents (*N* = 14, 10%) indicated that communities need to be informed ahead of time about the examination so that people could be prepared and available. Smaller numbers of respondents mentioned that the community would like to see their results from the last survey (*N* = 4, 3%). Importantly, a few leaders stated that the medicine promised after the last examination must come before the community would be willing to participate in further parasitological examinations (*N* = 4, 3%).

Again, there were differences between the districts. Respondents from Kolahun were less likely to think that researchers should expect a higher turnout than before (χ^2^(1) = 8.420, *P* = 0.004) and were more likely to think that that fear of EVD would keep people from participating in parasitological examinations (*P* = 0.002).

### Ebola's impact on study villages.

Nearly everyone (*N* = 138, 99%) said they knew about EVD. When asked to describe how someone can get EVD, 132 (94%) respondents named at least one of the transmission pathways emphasized by the Ministry of Health and World Health Organization: contact with a sick person (*N* = 125, 89.3%), contact with bushmeat (*N* = 46, 32.9%), or contact with a contaminated object (*N* = 3, 2.1%). Bushmeat was most often specified as monkey and bat. Respondents were confident that this information was widely known in their villages, with 136 (97%) answering that plenty of people or everyone knew how Ebola could be spread.

Fortunately, respondents did not report any cases of transmission of EVD within the DOLF study villages. However, survey respondents in two Foya District villages told us that some village members were diagnosed with EVD while traveling or working nearby. In Kilima, a border town about 3 km from Sierra Leone, several community members had traveled to Sierra Leone, where they became sick. Some died there, while one person received treatment and survived. In Ngorkuma, located 12 km north of Foya, a member of the community who worked at Foya Hospital contracted EVD and died after he cared for an infected woman who had crossed the border from Guinea. His daughter also died after contracting the disease. According to community health workers on the survey research team, who were involved in contact tracing during the outbreak, these were likely the first locally transmitted cases in Liberia. Neither the hospital worker nor his daughter stayed in Ngorkuma while they were ill.

Seventeen respondents told us that they had family members who had contracted EVD. All of the reported family cases of EVD occurred outside their home villages, mostly in Monrovia. A disproportionate number of these cases were in people whose home villages were in Kolahun District. Of respondents with infected family members, 41% (7 of 17) resided in Kolahun District where only 21% of the 140 interviews were performed. Only seven of twenty-six (27%) study villages in Foya District had respondents with affected family members compared with four of six (66%) study villages in Kolahun.

## Discussion

This study collected data about community perceptions of MDA for NTDs after the EVD outbreak in villages near an early epicenter of the outbreak. The results have important implications for NTD and other public health programs in the three countries severely affected by EVD. The most important and encouraging result was the continued enthusiasm for MDA and a willingness to continue participation in parasitological examinations after the outbreak of EVD. More than 90% of all the community leaders who participated in the survey felt that their community was ready for MDA and were personally willing to swallow the medications or participate in the examinations. In addition, respondents expressed high levels of community trust in the MDA programs. Even though 2 years had passed since the last round of MDA was delivered in some villages, respondents still credited the MDA program with improvements in health and were therefore willing to continue trusting the program. These overwhelming positive responses should bode well for MDA programs and suggest a strong support and desire among Liberians for the resumption of this public health program.

Although our results suggest that there is significant public support for the resumption of NTD control and elimination programs, they also suggest that programs should not assume that they can resume their routine activities as if nothing happened. Survey participants expected fewer people would comply with MDA when compared with the last distribution prior to the epidemic, voicing concerns from within the community about a link between MDA and EVD. Even as they stated that their villages were ready for MDA approximately one in three respondents suggested that fears about EVD would need to be overcome to improve coverage results. If these fears are not addressed when MDA programs resume, they may encounter lower than expected cooperation and compliance. This may be especially true in areas that were more directly affected by EVD.

Although there was no EVD transmission in our study villages, many people reported EVD cases in family members who lived outside their villages. This situation is likely to be typical for the majority of villages in the study area and nearby villages in Sierra Leone and Guinea. We encountered greater skepticism about MDA programs in Kolahun District, where a greater proportion of respondents reported having family members affected by EVD. Compared with Foya, respondents in Kolahun District were more concerned about making it clear that MDA and EVD are unrelated, and they expected a greater drop in coverage and participation in parasitological surveys because of fear of EVD. These results suggest that a positive relationship may exist between more direct encounters with EVD and suspicion that MDA programs could intentionally or inadvertently spread the disease.

Several themes emerged from our survey that point to strategies that could calm these fears and help MDA programs reengage communities and improve MDA compliance. Respondents were knowledgeable about the purpose of MDA and modes of EVD infection. Promoting this existing knowledge and their past experience with MDA could reassure people that MDA has nothing to do with EVD. Since past experiences with the MDA program seem to be the basis for a large part of the communities' trust in MDA, these programs should emphasize their past work. In particular, respondents thought that reminding the community that they have taken the same treatment before and that it was helpful would be a convincing message. Involving community members in these promotional messaging campaigns could be particularly effective. Previous research has shown how community involvement and ownership in MDA programs can achieve higher coverage rates, particularly in Africa.^10^,^11^ Our results underscore these findings, since including community members in MDA promotion activities was the most commonly suggested outreach method for improving MDA coverage. Many community leaders we surveyed seemed willing to promote MDA because they valued the collective benefits to the community. These motivations rooted in social norms that value duty and protecting the community can be important to an individual's decision to comply with MDA.^12^ Unlike many other health behavior campaigns that seek to motivate individuals, MDA is a community event that should be linked to the social behavior and identity of the group. Social norms played an important role in curbing the EVD outbreak in Liberia. Once the threat of EVD was understood, communities changed their burial practices, washed hands more frequently, and stopped shaking hands. Community leaders were often seen as the “driving force” of these initiatives.^13^ After the outbreak, the message should be that MDA is a similar protective action that requires everyone's participation to protect the community from disease. Leaders or other influential members of the community should once again play an important role in communicating these messages. This promotional messaging coupled with small practical changes to the MDA program (e.g., providing water in individual disposable paper cups for swallowing pills) should help to overcome fears surrounding MDA.

Our survey of community leaders quickly provided useful information related to the resumption of health programs that had been disrupted by the EVD outbreak. However, because of the limitation of the survey design the results should be interpreted with caution. The survey participants were a purposive sample of influential community leaders who could speak on behalf of the community. They were not a random sample of community members. Therefore, the results may not accurately represent opinions and sentiments of residents of the study villages. However, we attempted to include a diverse representation of community voices by including at least one youth and female leader in all but one village survey. Another limitation of our study was that it was conducted prior to resumption of MDA. Attitudes may change after MDA, and it would be interesting to conduct coverage surveys to determine how accurately participants' predictions matched with actual results.

With these limitations in mind, results of our study suggested that 32 communities in Foya and Kolahun districts were ready to resume MDA after the EVD outbreak, although a minority of survey participants suggested that there was fear in the community about a link between EVD and MDA. When MDA programs resume, they should address these fears early to avoid problems with compliance. This might be especially true in areas that experienced intense EVD transmission. Strategies to overcome these fears should emphasize the community's past experiences with MDA and enlist members of the community to promote and champion the program.

## Figures and Tables

**Table 1:** Number of survey participants by village

Foya District			
Bambuloe	4	Kpormbu	4
Bandenin	4	Langbamba	5
Chakporma	4	Lepaloe	4
Felaloe	6	Mendikorma	4
Fornin	4	Ngaisiakoryah*	4
Foya Dundu	4	Ngorkuma*	4
Gelema	4	Nongorchoe*	4
Kenema	4	Pehyama	5
Keyabendu	5	Sakawo	3
Kilima*	3	Sasanin	5
Kondobengu	5	Sayanin	4
Kpangbenin*	5	Sefedu	3
Kpelloe Ndama*	5	Wabengu*	5
Kolahun District			
Fokolahun*	4	Kporkulahun*	4
Kamatahun*	7	Yallahun	4
Kannela	4	Yandohun*	6

* Members of the community reported family cases of Ebola outside the village.

**Table 2:** Major survey results

Demographics	
Gender (*N* = 140)	
Male	103 (73.6%)
Female	37 (26.4%)
Average age	
All (*N* = 137)	47.3 (SD = 16.5)
Male (*N* = 102)	46.4 (SD = 17.1)
Female (*N* = 35)	49.8 (SD = 14.7)
Knowledge of MDA and Ebola	
What does MDA treat? (*N* = 140)*	
Filariasis	93 (66.4%)
Onchocerciasis	34 (24.3%)
Schistosomiasis	26 (18.6%)
Intestinal worms	21 (15.0%)
Malaria	10 (7.1%)
Other	9 (5.0%)
I do not know	8 (5.7%)
In your opinion how many people in your community know what the (MDA) medicine is for? (*N* = 140)	
Everyone	58 (41.4%)
Plenty of people	70 (50.0%)
Some people	7 (5.0%)
Few people	1 (2.1%)
No one	0 (0.0%)
Do not know	2 (1.4%)
How can you get Ebola? (*N* = 140)*	
Contact with a sick person	125 (89.3%)
Bushmeat (bat, monkey, etc.)	46 (32.9%)
Contact with a contaminated object	3 (2.1%)
Water	1 (0.7%)
I do not know	6 (4.3%)
In your opinion how many people in your community know how Ebola is spread? (*N* = 140)	
Everyone	92 (65.7%)
Plenty of people	44 (31.4%)
Some people	3 (2.1%)
Few people	0 (0.0%)
No one	0 (0.0%)
Do not know	1 (0.7%)
Trust in MDA programs	
Do people in your community trust the treatment program? (*N* = 131)	
Yes	130 (99.2%)
No	0 (0.0%)
I do not know	1 (0.8%)
Why did you answer this way?* (*N* = 131)	
The medicine was good, it improved health (generally)	118 (90.1%)
It improved onchocerciasis symptoms	22 (16.8%)
It improved schistosomiasis symptoms	6 (4.6%)
We know the people who provided MDA	10 (7.6%)
No one was hurt or suffered side effects	8 (6.1%)
We have been educated about MDA	7 (5.3%)
Perceived past and expected coverage	
In your opinion, how many people in your village swallowed the medicine pills given during the last treatment? (*N* = 140)	
4: everyone	50 (35.7%)
3: plenty of people	84 (60.0%)
2: some people	2 (1.4%)
1: few people	2 (1.4%)
0: no one	0 (0.0%)
MDA was not delivered in my village	1 (0.7%)
Do not know	1 (0.7%)
In your opinion, if the medicine was delivered to your community today, how many people would swallow the medicine? (*N* = 140)	
4: everyone	25 (17.9%)
3: plenty of people	65 (46.4%)
2: some people	22 (15.7%)
1: few people	13 (9.3%)
0: no one	4 (2.9%)
Do not know	11 (7.9%)
Do you think your community is ready for another round of MDA? (*N* = 140)	
Yes	130 (92.9%)
No	4 (2.9%)
I do not know	6 (4.3%)
Would you take the medication if MDA were delivered today? (*N* = 139)	
Yes	130 (93.5%)
No	9 (6.5%)

MDA = mass drug administration; SD = standard deviation.

* Categories are not exclusive; some respondents mentioned more than one category.

**Table 3:** Changes in coverage scores when asked to estimate number of people who swallowed the medicine at the last MDA and number who would swallow the medicine if it was delivered today using the following scale: 0 = no one, 1 = few people, 2 = some people, 3 = plenty of people, and 4 = everyone

Comparison of paired responses*	*N*
Expected coverage < past perceived coverage	52
Expected coverage > past perceived coverage	10
Expected coverage = past perceived coverage	66
Total	128

* Sign test: *z* = −5.207, *P* < 0.001; rejects H_0_ = participants are just as or more likely to expect positive changes as they are to expect negative changes; accepts H_a_ = more participants expect negative changes than positive changes.

**Table 4:** Community leader expectations regarding MDA and the research project by district

	Foya (*N* = 111)	Kolahun (*N* = 29)
	*n* (%)	*n* (%)
What do you think would convince more members of your community to comply with MDA and swallow or take the medicines?*		
Sensitize people before MDA (general education)†	93 (83.9)	19 (65.5)
Explain that the MDA is not Ebola related‡	15 (13.5)	9 (31.0)
Provide reassurance that procedures are similar to previous MDA	28 (25.2)	6 (20.7)
Use people from the village to share the message and educate the community	7 (6.3)	5 (17.2)
Use community health workers or GCHVs to educate people	3 (2.7)	2 (6.9)
Make a radio announcement	3 (2.7)	0 (0.0)
The drug distributors should take the medicine in front of the community	1 (0.9)	1 (3.4)
Nothing needs to be done	3 (2.7)	0 (0.0)
In your opinion, what issues do you expect the research team to face if they return to work in your community?*		
There will be no problems	50 (45.0)	13 (44.8)
More people will participate than before†	38 (34.2)	2 (6.9)
Few people will participate‡	8 (7.2)	9 (31.0)
The village needs to be told when the researchers are coming	9 (8.1)	5 (17.2)
The village needs to get past results before they participate in exams	4 (3.6)	0 (0.0)
The village needs to receive medicine before any examination	4 (3.6)	0 (0.0)
There are other problems that have been neglected besides filaria	1 (0.9)	0 (0.0)
Access to the village will be difficult	1 (0.9)	0 (0.0)

MDA = mass drug administration; GCHVs = general community health volunteers.

* Categories are not exclusive; some respondents mentioned more than one category.

† Significantly different at *P* < 0.05 level for χ^2^ test.

‡ Significantly different at *P* < 0.05 level for Fisher's exact test.
